# Unilateral Pulmonary Artery Lesion in an Immunocompromised Patient

**DOI:** 10.1002/rcr2.70778

**Published:** 2026-09-30

**Authors:** Seunghoon Lee, Kyung Soo Lee, Dong Young Jeong, Boram Lee, Junghee Lee, Dong Seop Jeong, Sung‐A Chang, Sun Hye Shin

**Affiliations:** ^1^ Department of Medicine, Samsung Medical Center Sungkyunkwan University School of Medicine Seoul Republic of Korea; ^2^ Department of Radiology, Samsung Medical Center Sungkyunkwan University School of Medicine Seoul Republic of Korea; ^3^ Department of Pathology and Translational Genomics, Samsung Medical Center Sungkyunkwan University School of Medicine Seoul Republic of Korea; ^4^ Department of Thoracic and Cardiovascular Surgery, Samsung Medical Center Sungkyunkwan University School of Medicine Seoul Republic of Korea; ^5^ Division of Cardiology, Department of Medicine, Samsung Medical Center Sungkyunkwan University School of Medicine Seoul Republic of Korea; ^6^ Division of Pulmonary and Critical Care Medicine, Department of Medicine, Samsung Medical Center Sungkyunkwan University School of Medicine Seoul Republic of Korea

**Keywords:** chronic thromboembolic pulmonary hypertension, immunocompromised host, pulmonary infarction, septic thrombus, unilateral pulmonary artery lesion

## Abstract

We report a 62‐year‐old man with myelodysplastic syndrome on immunosuppressive therapy who presented with right lung consolidation and neutropenia. Despite broad antimicrobial therapy for presumed pneumonia, negative microbiologic studies including bronchoalveolar lavage, and a nondiagnostic transbronchial biopsy, serial computed tomography (CT) scans showed progressive right‐sided consolidation, pleural effusion and CT signs of pulmonary hypertension. A routine contrast‐enhanced CT revealed a filling defect in the right pulmonary artery (PA). Dedicated CT pulmonary angiography confirmed persistent unilateral PA obstruction; together with pulmonary hypertension and a lack of improvement despite anticoagulation, this raised concern for chronic thromboembolic disease and PA malignancy. Fluorodeoxyglucose positron emission tomography (FDG PET) CT demonstrated mildly heterogeneous FDG uptake (SUV_max_ 2.9) favouring thrombus, and endobronchial ultrasound‐guided transbronchial needle aspiration yielded necrotic thrombotic material. Pulmonary endarterectomy, performed after interval clinical worsening, confirmed a chronic organizing septic thrombus. Weeks later, haemorrhagic infarction caused massive haemoptysis requiring wedge resection of the lung. This case highlights a rare septic thrombus in a unilateral PA leading to pulmonary infarction in an immunocompromised host.

## Graphical abstract

We report a case of a unilateral septic pulmonary artery thrombus presenting as pulmonary infarction and pulmonary hypertension in an immunocompromised host.

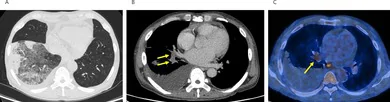

## 1 Introduction

Pulmonary infarction is uncommon, particularly in immunocompromised patients, because lung parenchymal lesions are frequently attributed to infection. Radiologic findings such as consolidation, ground‐glass opacity (GGO) and halo‐type nodules often prompt suspicion of bacterial pneumonia or invasive fungal disease, delaying recognition of vascular etiologies. We report a case in which persistent pulmonary consolidation in an immunocompromised host, initially attributed to pneumonia, proved to be pulmonary infarction with pulmonary hypertention caused by a unilateral septic pulmonary artery (PA) thrombus.

## 2 Case Report

A 62‐year‐old man with myelodysplastic syndrome (MDS) and marrow hypoplasia, previously treated with immunosuppressive therapy (rabbit anti‐thymocyte globulin and cyclosporine from June to October, 2024), underwent an outside non‐enhanced chest computed tomography (CT) that showed right lung consolidation. He had no fever or respiratory symptoms and had received empiric ciprofloxacin for presumed pneumonia without improvement. Upon presentation (Day 0), he was afebrile but neutropenic around this period (absolute neutrophil count 320–390/μL). Initial laboratory evaluation, including carcinoembryonic antigen, neuron‐specific enolase, β‐d‐glucan and Aspergillus antigen, was unremarkable.

A follow‐up non‐enhanced chest CT on Day 14 showed marked worsening of the right lower lobe (RLL) lesion, with mixed GGO and consolidation, and a new pleural effusion (Figure 1A); scattered nodules with a halo sign were also noted. Given the immunocompromised state and this radiologic pattern, invasive pulmonary aspergillosis was favoured, although mucormycosis could not be excluded. Around Day 14, a new cough developed, prompting admission; a single fever (38.1°C) was recorded around admission, with no other clinical signs of systemic infection. He never required supplemental oxygen during this hospitalization.

**FIGURE 1:**
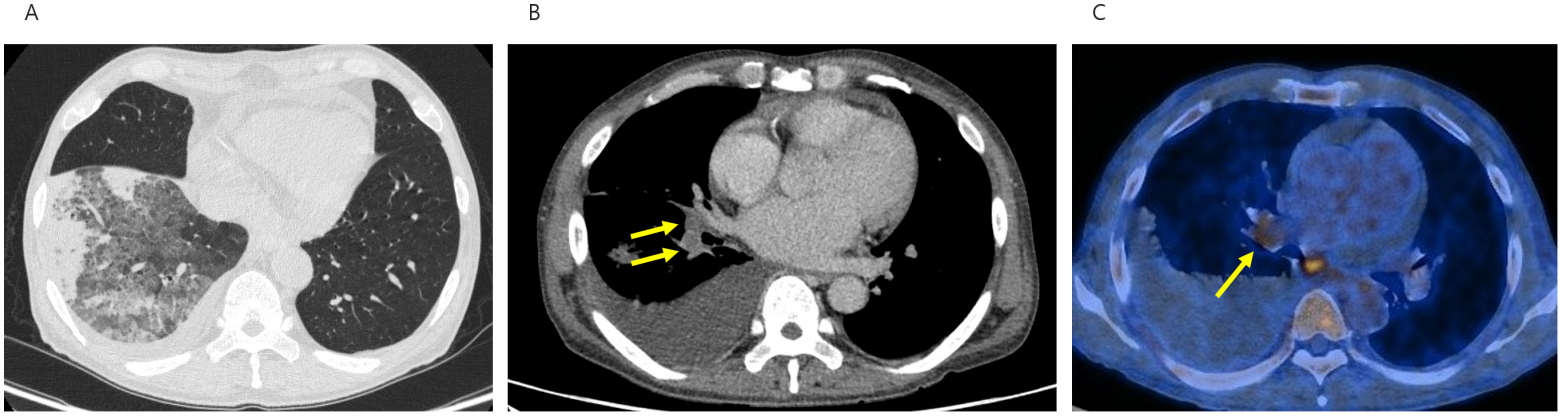
Radiologic and metabolic imaging in the diagnostic evaluation. (A) Non‐enhanced chest computed tomography (CT) on Day 14 showing mixed ground‐glass opacity and consolidation in the right lower lobe. (B) Contrast‐enhanced chest CT on Day 21 showing a low‐attenuation filling defect (arrow) in the right interlobar and lower lobar pulmonary artery. (C) Fluorodeoxyglucose positron emission tomography‐CT (PET‐CT) on Day 29 showing mildly heterogeneous FDG uptake (SUV_max_ 2.9, arrow) corresponding to the PA filling defect, favouring thrombus over sarcoma.

Bronchoscopy on Day 15 revealed no endobronchial lesions. Bronchoalveolar lavage (BAL) fluid from the right anterior basal segmental bronchus was xanthochromic; bacterial and fungal cultures, galactomannan and viral PCRs were all negative. Transbronchial lung biopsy demonstrated focal organizing pneumonia without fungal elements. Although he remained afebrile, liposomal amphotericin B and piperacillin/tazobactam were initiated for worsening parenchymal disease, with no radiologic improvement.

In the context of an initial focus on infection and coexisting chronic kidney disease, non‐enhanced chest CT was performed on Day 20. It demonstrated a dilated main PA, enlargement of the right‐sided cardiac chambers and pericardial effusion, suggestive of pulmonary hypertension (PH), prompting recommendations for echocardiography and contrast‐enhanced imaging. A routine contrast‐enhanced chest CT on Day 21 revealed a low‐attenuation filling defect in the right interlobar and RLL PAs with associated infarction, and anticoagulation was started (Figure 1B). Because this examination was not performed using a dedicated pulmonary embolism protocol, CT pulmonary angiography (CTPA) was obtained on Day 27 to better characterize the pulmonary arterial lesion. The filling defect persisted after 1 week of anticoagulation and extended into the lobar branches; its unilateral, expansile appearance raised concern for PA sarcoma.

FDG PET‐CT on Day 29 demonstrated mildly heterogeneous FDG uptake within the PA lesion (SUV_max_ 2.9), favouring thrombus over sarcoma (Figure 1C). On Day 31, endobronchial ultrasound (EBUS)‐guided transbronchial needle aspiration (TBNA) of the RLL PA yielded whitish necrotic tissue cores. Pathology reported predominantly thrombotic material with few atypical cells, which was insufficient to exclude malignancy. Right heart catheterization demonstrated precapillary PH, and angiography confirmed total occlusion of the RLL PA.

Given the persistent unilateral lesion despite nearly 1 month of anticoagulation, a low‐grade PA malignancy such as angiosarcoma remained a differential diagnosis, and pulmonary endarterectomy (PEA) was planned as both a therapeutic and diagnostic procedure. Because the patient remained clinically stable without an oxygen requirement or worsening dyspnoea at that time, PEA was scheduled electively rather than urgently. While awaiting surgery, he developed gradually worsening dyspnoea and increasing pleural effusion, prompting readmission; the operation was moved forward and performed on Day 87. At surgery, extensive material was identified within the right PA, and PEA was extended distally into the lobar branches, consistent with level III disease according to UCSD surgical classification (Figure 2A). Brown and Brenn staining revealed Gram‐positive cocci, confirming septic PA thrombus (Figure 2B). Postoperative bleeding required urgent surgical re‐exploration for haemostasis and hematoma evacuation, after which the patient recovered without further complications. The patient recalled a remote episode of dental implant infection, a potential source of bacteraemia and completed 4 weeks of ceftriaxone.

**FIGURE 2:**
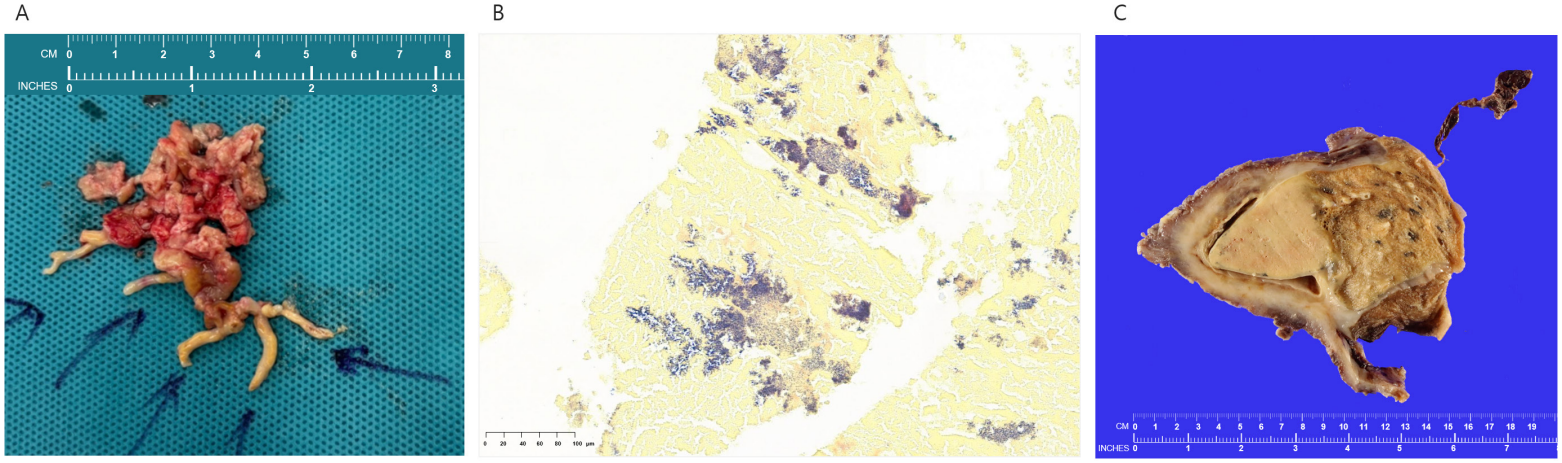
Surgical and pathological features of septic pulmonary artery thrombus and related complications. (A) Pulmonary endarterectomy specimen demonstrating level III disease (UCSD surgical classification of pulmonary endarterectomy disease levels). (B) Histopathology of the excised right pulmonary artery showing organizing thrombi containing bacterial colonies; Brown and Brenn stain reveals Gram‐positive cocci, confirming septic pulmonary thrombus. (C) Gross photograph of the wedge‐resected lung specimen obtained by video‐assisted thoracoscopic surgery, showing extensive haemorrhagic necrosis.

Six weeks later, he developed massive haemoptysis due to haemorrhagic infarction and underwent emergency wedge resection, which revealed extensive haemorrhagic necrosis (Figure 2C). He remained clinically stable thereafter until the MDS underwent leukaemic transformation.

## 3 Discussion

This case reports a rare presentation of unilateral septic PA thrombus leading to pulmonary infarction in an immunocompromised host, which mimics pneumonic consolidation and PA sarcoma. Although imaging initially suggested pneumonia, the clinical course was inconsistent with infection. Apart from a single fever around the time of admission, systemic infectious features were absent, and repeated microbiologic studies remained negative. Xanthochromic BAL fluid, in retrospect, provided an early clue to occult alveolar haemorrhage related to pulmonary infarction [1]. In addition, early CT findings of PH and peripheral infarction supported a vascular rather than infectious aetiology.

Unilateral PA obstruction encompasses a broad differential diagnosis, including pulmonary thromboembolism, non‐thrombotic embolism, PA tumours and immune‐mediated vasculopathies [2]. Persistent obstruction despite anticoagulation should prompt consideration of chronic thromboembolic pulmonary hypertension (CTEPH) or PA sarcoma. CTEPH typically shows vessel narrowing or intraluminal webs on CT, whereas PA sarcoma is exceptionally rare, with an estimated incidence of 0.001%–0.03% [3], and appears as an expansile, lobulated intraluminal mass on CT.

In this patient, persistent unilateral obstruction despite anticoagulation and precapillary PH supported a chronic process. As CT findings were not sufficiently specific to reliably distinguish CTEPH from PA sarcoma, FDG PET‐CT and EBUS‐TBNA of the PA lesion, the latter providing safe tissue acquisition [4], were pursued to aid diagnosis. Mild FDG uptake favoured thrombus over sarcoma, and EBUS‐TBNA yielded necrotic thrombotic material with rare atypical cells, which was non‐diagnostic, ultimately requiring surgical pathology.

A distinctive feature was the septic PA thrombus without classic signs of sepsis or infective endocarditis; septic pulmonary embolism can present with attenuated systemic manifestations, particularly in patients with prior antimicrobial exposure or immunosuppression, delaying recognition [5]. In this case, prior antibiotics and immunosuppression may have masked systemic inflammation, while a remote dental implant infection was considered a possible source.

This case highlights several learning points for similar patients. Pulmonary consolidation with an atypical course or poor antimicrobial response should prompt consideration of pulmonary infarction, and earlier dedicated CTPA should be considered once a vascular aetiology is suspected. Persistent unilateral PA filling defects despite adequate anticoagulation, especially with PH, warrant evaluation for chronic thromboembolic disease and PA malignancy. PET‐CT and EBUS‐TBNA can provide useful clues but may remain non‐diagnostic, and surgical biopsy or endarterectomy may ultimately be required. Septic organizing thrombus remains a rare but relevant differential diagnosis in immunocompromised hosts.

## Author Contributions

**Seunghoon Lee:** writing – original draft, data curation, investigation, visualization. **Kyung Soo Lee:** visualization, writing – review and editing, methodology, supervision. **Dong Young Jeong:** visualization, writing – review and editing, methodology. **Boram Lee:** methodology, visualization, writing – review and editing. **Junghee Lee:** writing – review and editing. **Dong Seop Jeong:** writing – review and editing, supervision. **Sung‐A Chang:** supervision, writing – review and editing, investigation. **Sun Hye Shin:** investigation, methodology, data curation, supervision, resources, writing – review and editing, writing – original draft.

## Funding

The authors have nothing to report.

## Ethics Statement

This study was approved by the Institutional Review Board of Samsung Medical Center (IRB no. SMC 2026‐06‐128).

## Consent

The authors declare that written informed consent was obtained from the patient's next of kin for the publication of this manuscript and accompanying images using the consent form provided by the Journal.

## Conflicts of Interest

The authors declare no conflicts of interest.

## Data Availability

The data that support the findings of this study are available on request from the corresponding author. The data are not publicly available due to privacy or ethical restrictions.
